# 810. Cardiac Pacemaker Implantation Surgery: Automated Prediction of Surgical Site Infection

**DOI:** 10.1093/ofid/ofab466.1006

**Published:** 2021-12-04

**Authors:** Flávio Henrique Batista de Souza, Bráulio R G M Couto, Felipe Leandro Andrade da Conceição, Gabriel Henrique Silvestre da Silva, Igor Gonçalves Dias, Rafael Vieira Magno Rigueira, Gustavo Maciel Pimenta, Maurilio B Martins, Júlio César O Mendes, Guilherme Brangioni Januário, Rayane Thamires Oliveira, Laura Ferraz de Vasconcelos, Laís L de Araújo, Ana Clara Resende Rodrigues, Camila Morais Oliveira E Silva, Eduarda Viana De Souza, Júlia Faria Melo, Maria Cláudia Assunção De Sá, Walquíria Magalhães Silva, Gabriella Mourão Cerqueira Figueiredo, Jamile Doffénond Colares, Ludmila Paula Guimarães Junqueira, Maiara Cristina Reis, Mariana Ribeiro Nobre

**Affiliations:** 1 Centro Universitário de Belo Horizonte, Belo Horizonte, Minas Gerais, Brazil; 2 Centro Universitário de Belo Horizonte - UniBH, Belo Horzonte, Minas Gerais, Brazil

## Abstract

**Background:**

A research focused on surgical site infection (SSI) was performed in patients undergoing cardiac pacemaker implantation surgery. The main objective is to statistically evaluate such incidences and enable a study of the prediction power of SSI through pattern recognition algorithms, in this case the Multilayer Perceptron (MLP).

**Methods:**

Data were collected from five hospitals in the city of Belo Horizonte (more than 3,000,000 inhabitants), between July 2016 and June 2018, on SSI by the Hospital Infection Control Committees (CCIH) of the hospitals involved in the search. All data used in the analysis during their routine SSI surveillance procedures were collected. So, three procedures were performed: a treatment of the collected database for use of intact samples; a statistical analysis on the profile of the hospitals collected and; an assessment of the predictive power of five types of MLP (Backpropagation Standard, Momentum, Resilient Propagation, Weight Decay, and Quick Propagation) for SSI prediction. MLPs were tested with 3, 5, 7, and 10 hidden layer neurons and a database split for the resampling process (65% and 75% for testing, 35% and 25% for validation). They were compared by measuring AUC (Area Under the Curve - from 0 to 1) presented for each of the configurations.

**Results:**

From 1394, 572 records were: 21% of deaths and 2.4% patients had SSI; from the confirmed SSI cases, approximately 64.3% had sites classified as “clean”; length of hospital stay ranged from 0 to 175 days (from 1 to 70 days); the average age is 67 years. The prediction power of SSI, the experiments achieved from 0.409 to 0.722.

**Conclusion:**

Despite the considerable loss rate of more than 65% of the database samples due to the presence of noise, it was possible to have a relevant sampling for the profile evaluation of Belo Horizonte hospitals. Moreover, for the predictive process, although some configurations reached 0.722. To optimize data collection and enable other hospitals to use the SSI prediction tool (available in www.nois.org.br ), two mobile application were developed: one for monitoring the patient in the hospital and the other for monitoring after hospital discharge.

**Disclosures:**

**All Authors**: No reported disclosures

